# An Integrated Testbed for MITRE-Mapped Attack Emulation in Industrial Control Networks †

**DOI:** 10.3390/s26113514

**Published:** 2026-06-02

**Authors:** Jaafer Rahmani, Kai Oliver Detken, Axel Sikora

**Affiliations:** 1 Institute of Reliable Embedded Systems and Communication Electronics (ivESK), Offenburg University of Applied Sciences, 77652 Offenburg, Germany; axel.sikora@hs-offenburg.de; 2 Faculty of Engineering, University of Freiburg, 79110 Freiburg, Germany; 3 DECOIT GmbH & Co. KG, 28359 Bremen, Germany; detken@decoit.de

**Keywords:** Industrial Control Systems, Operational Technology, testbed, anomaly detection, autoencoder, CALDERA, multi-stage attack emulation, MITRE ATT&CK, Modbus, PROFINET

## Abstract

Evaluating intrusion detection methods at the level of individual MITRE Adversarial Tactics, Techniques, and Common Knowledge (ATT&CK) for Industrial Control System techniques requires Operational Technology traffic in which each attack sequence carries its MITRE technique identifier as ground truth. Publicly available Industrial Control System datasets either provide coarse attack-versus-benign labels (SWaT, WADI, CIC-APT-IIoT) or require ex-post technique reconstruction from CALDERA operation logs, and therefore do not support per-technique benchmarking. We describe one primary contribution and two supporting contributions, demonstrated on one Modbus/Raspberry-Pi programmable logic controller/CALDERA/convolutional bidirectional Long Short-Term Memory autoencoder (CNN-BiLSTM-AE) use case. The *primary* contribution is an in-orchestrator labelling methodology for per-technique-labelled Industrial Control System attack capture. Its single load-bearing property is that the campaign orchestrator owns the label primitive and writes each per-sequence technique identifier into the capture artefact at injection time, eliminating ex-post log-to-packet alignment. The *first supporting* contribution is a protocol-aware detection pipeline. Its load-bearing architectural choice is a priority-ordered protocol router that dispatches each labelled flow to a per-protocol detector plug-in (protocol-aware features here, with generic-flow features admissible as an alternative plug-in policy on the same router). The *second supporting* contribution is a suite of four reproducible CALDERA chains (three Information-Technology-to-Operational-Technology kill chains plus one enterprise-side control) that exercise the labelling methodology end-to-end and the detection pipeline along complementary detection paths. All three contributions are platform-independent: any ATT&CK-aligned emulator and any fieldbus protocol can host the labelling methodology, and any detector trained on an admissible feature space can plug into the router. The dataset contains 40,000 benign and 9997 attack Modbus sequences spanning four ATT&CK techniques (T0802 Automated Collection, T0831 Manipulation of Control, T0836 Modify Parameter, T0846 Remote System Discovery). On this dataset, the CNN-BiLSTM-AE reaches a 100% true-positive rate (TPR) at the 98th-percentile benign threshold across all four techniques and a 99.7% overall TPR at the tighter 99.5th-percentile threshold, with per-technique TPR between 96.1% (T0836 Modify Parameter) and 100% (T0802 Automated Collection, T0846 Remote System Discovery). Across the four CALDERA chains, the Modbus autoencoder produces 234 protocol-layer detections and the Security Information and Event Management (SIEM) rule set produces 30 alerts, with per-chain tactic coverage between 0.714 and 0.786 and CALDERA-ability success rates between 0.800 and 0.857.

## 1. Introduction

Industrial Control Systems (ICSs) are foundational to critical infrastructure sectors including energy production, manufacturing, water treatment, and transportation [1]. These systems increasingly converge with enterprise Information Technology (IT) networks through Industrial Internet of Things (IIoT) architectures, expanding the attack surface available to adversaries [2]. Legacy fieldbus protocols such as Modbus/TCP and PROFINET, designed for reliability in isolated environments, lack encryption and authentication mechanisms, making them vulnerable to reconnaissance, manipulation, and denial-of-service attacks [3,4]. Modern threat actors target industrial environments through multi-stage campaigns that combine IT-domain techniques (credential theft, lateral movement) with OT-domain actions (process manipulation, safety system tampering); the MITRE ATT&CK for Industrial Control System framework documents over 80 such techniques across 12 tactics [5].

We address the per-technique evaluation gap with an integrated testbed that combines a physical OT network, multi-layer monitoring (network flows, host events, protocol transactions), MITRE CALDERA [6] adversary emulation, and an in-orchestrator labelling path that writes per-sequence MITRE ATT&CK for ICS technique identifiers into the captured traffic at injection time.

This article extends our earlier conference paper [7], which introduced the physical OT testbed for cyber-attack emulation and AI-driven anomaly detection; the present article develops the in-orchestrator MITRE ATT&CK for ICS labelling methodology, the protocol-aware detection pipeline, and the per-technique evaluation reported here.

This paper makes one primary contribution and two supporting contributions, all demonstrated on one operational use case on a physical OT testbed.

The *primary* contribution is an in-orchestrator labelling methodology. Its single *load-bearing property* (the one property on which the contribution stands or falls) is that the campaign orchestrator owns the label primitive. Each per-sequence technique identifier is written into the capture artefact at injection time by the agent that knows which technique it is executing. Technique-to-traffic attribution therefore does not depend on aligning operation logs against packet timestamps after the fact. The labelling interface is portable to other ATT&CK-aligned emulators. What is novel relative to SWaT, WADI, and CIC-APT-IIoT is per-technique ATT&CK-for-ICS ground truth in the capture itself; what is novel relative to existing CALDERA-for-ICS deployments is that the label is written at injection time rather than reconstructed ex post.

The *first supporting* contribution is a protocol-aware detection pipeline that consumes the labelled corpus. Its load-bearing architectural choice is a *priority-ordered protocol router*: a classifier whose rules are applied top-down so the most specific rule a flow matches wins. The router dispatches each labelled flow to a per-protocol detector plug-in. Several feature-engineering policies are *admissible* at this plug-in interface (the term names any feature vector whose input shape matches the deployed detector’s training-time input): protocol-aware features, generic flow features, or any other admissible feature space. The labelling primitive is therefore not coupled to a particular detector. What is novel relative to Kitsune-style ensemble autoencoders is that each flow is scored in a protocol-specific feature space rather than in a single homogeneous one.

The *second supporting* contribution is the four reproducible CALDERA chains that exercise the labelling methodology end-to-end (Section 4) and the pipeline along complementary detection paths (Section 6.3).

A practitioner running, for example, Atomic Red Team plus DNP3 plus a generic-flow detector can apply the labelling methodology and the router architecture unchanged. The Modbus/Raspberry-Pi-PLC/CALDERA/CNN-BiLSTM-AE combination reported here is the form of the evidence we offer for the three contributions, not a constraint on their scope.

The remainder of this paper is organised as follows. Section 2 reviews related testbed architectures, attack emulation platforms, and detection approaches, and closes (Section 2.4) by stating the contributions of this paper against the three gaps identified there. Section 3 describes the testbed architecture and multi-layer monitoring infrastructure. Section 4 presents the attack emulation framework. Section 5 describes the protocol-aware detection pipeline. Section 6 reports the per-technique evaluation on the MITRE-mapped Modbus attack dataset and the multi-path validation across four CALDERA chains. Section 7 discusses findings and limitations, and Section 8 concludes.

## 2. Related Work

Prior work on industrial-control-system testbeds and detection sits in three threads: (i) testbed infrastructure (hardware, protocols, monitoring), (ii) adversary emulation that generates attack traffic, and (iii) anomaly-detection methods that consume the traffic. Each subsection surveys one thread.

### 2.1. Industrial Control System Testbeds

Research testbeds for ICS security span single-protocol platforms (Hahn et al. [8] on smart-grid cyber-physical co-simulation; de Brito and de Sousa [9] on Modbus for nuclear power), multi-protocol or multi-process frameworks (CPGrid-OT [10] on DNP3, ICSSIM [11] with virtualised processes, Noorizadeh et al. [12] for chemical false-data injection), and multi-stage attack benchmarks (SWaT and WADI [13] for water treatment, CIC-APT-IIoT [14] for provenance-based APT traces). These platforms vary in protocol breadth and attack-vector coverage but share a common label granularity: process-state, attack-versus-benign, or session-level labels, none aligned with the MITRE ATT&CK for ICS technique taxonomy.

**Gap.** Existing ICS testbeds either fix on a single protocol family (Hahn et al., de Brito and de Sousa, CPGrid-OT) or, when broader in scope, emit attack traffic with labels that are not aligned with the MITRE ATT&CK for ICS technique taxonomy: SWaT and WADI carry process-state labels for water-treatment-specific scenarios, and CIC-APT-IIoT carries provenance-based attack-versus-benign labels without per-technique identifiers. No prior testbed in this thread produces network traffic with per-MITRE-technique ground truth written at injection time.

### 2.2. Adversary Emulation and Attack Frameworks

MITRE CALDERA [6] automates the execution of ATT&CK-aligned attack campaigns through Sandcat agents (managed implants installed on target hosts that report back over HTTPS) which replay named *abilities* (the action units composing a campaign profile); the CALDERA-for-ICS plugin extends this to OT attack techniques. The MITRE ATT&CK for ICS framework [5] provides the tactic-technique taxonomy used to label attack activity in supervised evaluation.

**Gap.** Existing CALDERA-for-ICS deployments execute MITRE-aligned campaigns on OT testbeds but do not write the technique identifier of each emitted action into the captured network traffic; technique-to-traffic attribution must be reconstructed ex post by aligning CALDERA operation logs with packet timestamps, which loses fidelity when multiple abilities overlap in time or when traffic capture and orchestrator clocks drift.

### 2.3. Anomaly Detection for Industrial Control Systems

Machine learning approaches to ICS anomaly detection span supervised classifiers trained on labelled attack data [15] and unsupervised methods that learn normal operational patterns. Autoencoders are widely adopted for the latter: Mirsky et al. [16] proposed Kitsune, an ensemble of autoencoders for online network intrusion detection. For ICS environments, autoencoders trained on protocol-specific features (Modbus function codes, register values, timing patterns) can detect anomalies that generic network flow models miss.

The challenge of protocol diversity in ICS environments motivates protocol-aware detection. A Modbus anomaly detector must understand function codes and register addressing, while a PROFINET detector must analyse cyclic timing and inter-arrival-time (IAT) patterns. Hamolia et al. [17] demonstrated that latent-space representations combined with machine learning improve detection in high-dimensional network data, a principle that applies to multi-protocol ICS environments where feature spaces differ across protocols.

**Gap.** Kitsune-style ensemble autoencoders [16] and the majority of subsequent ML-based ICS detectors operate on a single homogeneous feature space, which forces a lowest-common-denominator representation across protocols and rules out per-protocol features such as Modbus function-code and register-address structure or PROFINET cycle-timing statistics.

### 2.4. Contributions of This Extension

The three gaps identified in Section 2.1, Section 2.2 and Section 2.3 motivate the contributions of this paper. The primary contribution is the in-orchestrator labelling methodology; the two supporting contributions describe the testbed campaigns that exercise it and the detection pipeline that consumes its output.

1. **Primary: In-orchestrator MITRE ATT&CK for ICS labelling methodology.** Each emitted attack sequence is tagged with its MITRE technique identifier by the campaign orchestrator at injection time, not inferred ex post from captured traffic. This separates the proposed methodology from publicly available ICS datasets such as SWaT and WADI [13] (process-state labels tied to water-treatment scenarios, no per-technique ATT&CK mapping), CIC-APT-IIoT [14] (provenance-based attack-versus-benign labels without MITRE technique identifiers), and from existing CALDERA-for-ICS deployments that emit ATT&CK-aligned campaigns but require ex-post correlation of operation logs with captured traffic to recover technique attribution. This closes the labelling gap of Section 2.1: writing per-MITRE-technique ground truth at injection time is the testbed capability that gap identifies as absent from prior work. Applied to Modbus, the methodology yields the per-technique labelled corpus of 9997 attack sequences on which the evaluation of Section 6 is run.
2. **Supporting: Reproducible multi-stage CALDERA campaigns.** Four chain profiles (three IT-to-OT kill chains plus one IT-only enterprise-side control) are defined as YAML campaign profiles and executed end-to-end with per-chain ground-truth labels written by the orchestrator (Section 4). The chains exercise the labelling methodology across credential access, lateral movement, and protocol-level OT manipulation, providing the multi-stage validation that single-technique benchmarks do not, and closing the technique-to-traffic attribution gap of Section 2.2.
3. **Supporting: Protocol-aware detection pipeline.** A priority-ordered protocol router dispatches each flow to the protocol-specific detector applicable to it (Section 5). Compared to Kitsune-style ensemble autoencoders [16] that operate on a single homogeneous feature space, the router enables per-protocol feature engineering (Modbus function codes and register addresses; PROFINET inter-arrival-time patterns) while sharing the same labelled-evaluation interface produced by the primary contribution, closing the feature-space gap of Section 2.3.

**Scope of the evidence offered.** The contributions above describe one operational use case of the primary contribution (the labelling methodology, with one load-bearing property) and its two supporting contributions: the reproducible CALDERA chains that exercise the labelling methodology end-to-end, and the protocol-aware detection pipeline (with one load-bearing architectural choice) that consumes the labelled corpus. The labelling primitive is portable to other ATT&CK-aligned emulators (Atomic Red Team, MITRE Engenuity Center for Threat-Informed Defense) by re-implementing the per-sequence write at the equivalent point in the campaign profile. The protocol router admits per-protocol detector plug-ins trained on any admissible feature space: protocol-aware features here (Modbus function codes, register addresses, inter-arrival-time statistics) and generic-flow features (NetFlow five-tuple aggregates, byte and packet counts) are two coherent plug-in policies on the same router architecture, and the choice between them is a feature-engineering decision at the plug-in interface; adopting a different plug-in alters neither the methodology nor the router. Subsequent sections describe and evaluate the Modbus use case; the per-technique results in Section 6 therefore report the behaviour of the methodology and pipeline on this one instance of the use-case protocol.

## 3. Testbed Architecture

**Figure 1:**
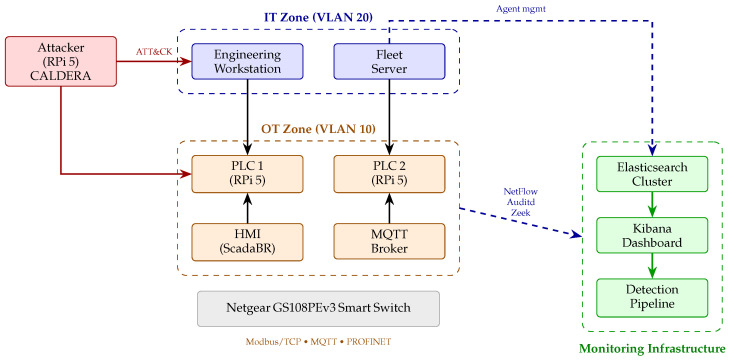
Testbed architecture. Read left-to-right in three zones: (i) the attacker node (left) runs MITRE CALDERA and emits ATT&CK-aligned abilities; (ii) the monitored network (centre, shaded) comprises an IT zone above and an OT zone below, separated by a VLAN boundary on a managed switch; (iii) the monitoring infrastructure (right) collects multi-layer telemetry (network flows, host events, Modbus and PROFINET protocol transactions) via Elastic Agents and forwards it to the detection pipeline (Section 5) and the SIEM. Solid arrows mark attack execution paths; dashed arrows mark telemetry-collection paths.

The testbed emulates a realistic ICS environment by integrating physical OT devices with virtualised IT components, connected through a segmented network with multi-layer monitoring. Figure 1 illustrates the architecture.

### 3.1. Operational Technology Network

The OT network comprises the following physical and virtualised components:

- **Programmable Logic Controllers:** Two Raspberry Pi 5 devices (Raspberry Pi Ltd., Cambridge, UK) serve as Modbus server/client pairs on port 502; running Linux admits the same Elastic Agent/Auditd/NetFlow telemetry stack as the IT nodes, giving unified host-layer visibility. Industrial PLC timing fidelity is treated in Section 7.4.
- **Human–Machine Interface:** A ScadaBR virtual machine (open-source, native Modbus/TCP) provides Supervisory Control and Data Acquisition (SCADA) visualisation and control over the PLC pair.
- **MQTT Broker:** A Message Queuing Telemetry Transport (MQTT) broker equipped with Zeek [18] monitors telemetry topics and forwards protocol-level events to the SIEM.

### 3.2. Information Technology Network

- **SIEM Infrastructure:** An Elastic Stack 8.19 deployment (Elasticsearch, Logstash, Kibana; Elastic N.V., San Francisco, CA, USA) serves as the central Security Information and Event Management system.
- **Engineering Workstation:** A Windows Server virtual machine functions as the administrative hub, replicating the configuration and management capabilities of enterprise ICS deployments.
- **Attacker Node:** A dedicated Raspberry Pi 5 runs the MITRE CALDERA framework with OT-specific plugins, executing automated attack campaigns against both IT and OT network segments.

### 3.3. Network Segmentation and Monitoring

A Netgear GS108PEv3 smart switch (NETGEAR, Inc., San Jose, CA, USA) implements Virtual Local Area Network (VLAN) segmentation, separating IT and OT network zones. This segmentation enables the study of lateral movement and VLAN hopping attack vectors while maintaining realistic network architecture.

The multi-layer monitoring system captures telemetry across three dimensions:

- **Network layer (NetFlow):** Source/destination IP addresses, ports, flow duration, packet and byte counts, and flow direction for all inter-device communications.
- **Host layer (Auditd):** Process execution, file access and modification, authentication events, privilege changes, and security alerts on all monitored Linux devices.
- **Protocol layer (Zeek):** Deep packet inspection of Modbus transactions (function codes, register addresses, request/response payloads), MQTT topic activity, HTTP/HTTPS traffic, and DNS queries.

All monitoring data is time-synchronised and indexed in Elasticsearch, enabling cross-layer event correlation across the network, host, and protocol layers.

## 4. Attack Emulation Framework

The attack emulation framework drives the orchestrator’s labelling path that produces the MITRE-mapped Modbus dataset of Section 6 and the multi-stage chain telemetry of Section 6.3. Four chains are evaluated so that the suite exercises complementary detection paths (ML+SIEM, ML-dominant, ML-only, SIEM-only) and each layer’s coverage is independently inspectable. CALDERA is selected over hand-written attack scripts and ICS-specific frameworks (e.g., GRFICS): its declarative ATT&CK-mapped abilities make the technique-to-action mapping auditable, Sandcat agents support deterministic ability-sequence replay across runs, and the campaign-profile YAML is itself the experimental record.

### 4.1. CALDERA Integration

MITRE CALDERA 5.3.0 (The MITRE Corporation, McLean, VA, USA) is deployed on a dedicated attacker node with the CALDERA-for-ICS plugin, enabling OT-specific attack techniques alongside the standard enterprise plugin set.

Four chain profiles are defined in YAML and executed end-to-end. Each chain combines enterprise and ICS-domain abilities to exercise a different detection path; tactic and technique mappings follow the abilities the chain executes, and the ability success rate per chain is reported alongside the detection counts in Section 6.3.

1. **Chain 1 (phish + lateral + Modbus write):** An 11-ability chain combining initial access via phishing, IT lateral movement, and a Modbus write payload. Techniques: T1110.003 (Password Spraying), T1021.002 (Server Message Block (SMB)/Windows Admin Shares), T0888 (Remote System Information Discovery), T0861 (Point & Tag Identification), T0836 (Modify Parameter), T0806 (Brute Force I/O).
2. **Chain 2 (password spray + persistence to OT):** An 11-ability chain emphasising credential abuse and persistence (T1110.003 Password Spraying, T1098 Account Manipulation), traversing into OT discovery and modification abilities (T0888 Remote System Information Discovery, T0861 Point & Tag Identification, T0836 Modify Parameter).
3. **Chain 3 (Linux credential theft, PLC target):** A 5-ability chain originating on a Linux node, harvesting credentials (T1003 OS Credential Dumping) and pivoting to a PLC (T0888 Remote System Information Discovery, T0836 Modify Parameter).
4. **Chain 4 (domain escalation via Group Policy Object (GPO)):** A 14-ability enterprise-focused chain (T1087.002 Domain Account Discovery, T1482 Domain Trust Discovery, T1484.001 Group Policy Modification, T1098 Account Manipulation) whose actions stay on the IT side of the network. Chain 4 produces no Modbus-protocol detections by design and exercises the SIEM rule set independently of the ML path.

### 4.2. Campaign Orchestrator

In addition to CALDERA-based profiles, a Python 3.12-native campaign orchestrator provides fine-grained control over Modbus attack primitives. The orchestrator implements five campaign types:

- **Full kill chain:** Reconnaissance → Initial Access → Collection → Evasion → Impact, executing 21 MITRE ICS techniques in sequence.
- **Reconnaissance:** Device identification (FC 43 MEI: the Modbus Encapsulated Interface Function Code 43, which Modbus servers expose to publish device-identification metadata such as vendor, product code, and revision), register scanning across address ranges, and function-code probing to map available services.
- **Stealth:** Low-rate reconnaissance and collection with inter-request delays designed to evade threshold-based detection.
- **Denial of service:** Sustained 1000-request floods targeting Modbus services, exceeding typical SCADA polling rates (1–10 req/s) by two to three orders of magnitude.
- **Impact only:** Direct register manipulation and coil forcing without prior reconnaissance.

Each campaign generates a ground-truth JSON Lines (JSONL) file containing per-attack labels with fields for stage, technique identifier, expected detection response, and attack intensity. This structured labelling supports both supervised and unsupervised evaluation of detection models.

### 4.3. Attack Primitives

The Modbus attack implementation covers the following primitives, each mapped to MITRE ATT&CK for ICS techniques:

- **Register scanning** (T0846): Sequential reads across register address ranges to discover active data points.
- **Coil manipulation** (T0806): Forced output writes to discrete control points.
- **Rapid polling/DoS** (T0814): High-frequency read requests exceeding normal polling rates.
- **Invalid protocol identifiers** (T0831): Modbus Application Protocol (MBAP) headers with non-standard protocol IDs (e.g., 0x1337) to test parser robustness.
- **Illegal function codes**: Requests with function codes outside the Modbus specification (e.g., 0x80) to probe error handling.
- **Broadcast writes** (T0813): Write requests with unit ID 0 targeting all devices on the bus.
- **Transaction ID mismatch**: Responses with mismatched transaction IDs to test session tracking.
- **Device identification** (T0888): MEI (Modbus Encapsulated Interface) FC 43 requests to extract device metadata.

#### Artefacts That Specify the Experiment

The orchestrator emits two artefacts per run that together specify the experiment, so that an independent execution is fully constrained without reading the orchestrator source code. The first is a YAML campaign profile that names the ability sequence, the MITRE technique identifier of each ability, and the per-ability parameters (target host, register range, payload, inter-request delay). The second is a per-sequence JSONL ground-truth file whose record schema is {stage, technique_id, expected_response, intensity, timestamp}. Executing the same profile on the same testbed reproduces the same labelled sequence set up to network-level jitter. The raw packet capture and the per-technique labelled corpus are subject to the consortium non-disclosure agreement recorded in the Data Availability Statement and are not publicly redistributable; an independent reproduction against a comparably equipped testbed is the supported reproduction path, using the YAML profile schema and JSONL record schema described above together with the four CALDERA chain definitions of Section 4.

## 5. Detection Pipeline

**Figure 2:**
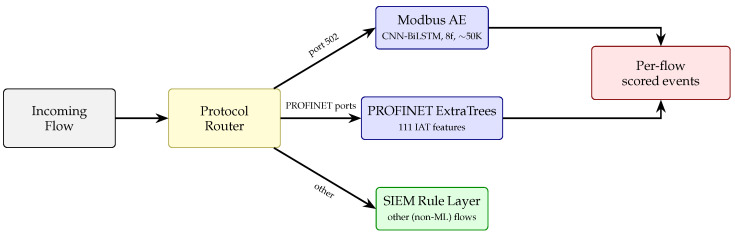
Detection pipeline architecture. The protocol router classifies each incoming flow and dispatches it one of three ways: Modbus flows to the CNN-BiLSTM autoencoder, PROFINET flows to the ExtraTrees detector, and all other flows to the SIEM rule layer, bypassing the machine learning stage. The two machine learning detectors produce per-flow scored events, consumed by a downstream SIEM-based multi-layer collection-and-detection framework. All arrows denote the direction of flow through the pipeline.

The detection pipeline processes incoming network flows through two stages: protocol classification, followed by dispatch to the protocol-specific detector. This separates Modbus (request–response on variable-latency TCP) from PROFINET (cyclic real-time at tight inter-arrival intervals), avoiding the over- or under-fit tradeoff of a single universal detector on pooled traffic. Figure 2 illustrates the pipeline architecture.

### 5.1. Protocol Router

The protocol router classifies each incoming flow into one of three categories: modbus, profinet, or other. Only modbus and profinet flows reach the ML detectors; flows classified as other (IT traffic, HTTP, MQTT, reconnaissance activity) bypass the ML stage and are routed to the SIEM rule layer.

Classification follows a priority-ordered rule set. The order is not arbitrary: each rule is strictly more specific than the rules below it, so applying them top-down produces the most restrictive classification a flow qualifies for. Reconnaissance tokens take precedence over port-based classification because scan traffic frequently targets port 502 with zero Modbus-protocol payload, and a port-first dispatch would waste Modbus-detector capacity on flows that are definitionally not Modbus. Explicit metadata hints come second because upstream parsers (Zeek, Elastic integrations) provide protocol labels that are stronger evidence than a port number alone. Port-based classification comes third because ports are standard but not exclusive (port 502 can carry non-Modbus traffic in misconfigured deployments). The PROFINET branch additionally gates on packet count: a session carrying ≤3 packets cannot evidence the cyclic inter-arrival structure that defines PROFINET traffic at 1–10 ms frame rates, so such low-packet flows on PROFINET ports are reclassified as scan activity rather than fed to a timing-based detector on too few samples. The priority rules are as follows:

1. Flows containing reconnaissance tokens (scan, probe, recon) are classified as other regardless of port.
2. Explicit protocol hints in metadata override port-based classification.
3. Flows on TCP port 502 are classified as modbus.
4. Flows on PROFINET ports (34962, 34963, 34964, 102) are classified as profinet, with low-packet-count flows (≤3 packets) reclassified as scan activity.
5. Common application ports (80, 443, 8080) are classified as other.
6. Default: other.

For batch processing, majority voting across samples determines the batch protocol classification.

### 5.2. Modbus Anomaly Detector

The Modbus detector is an autoencoder: a neural model trained only on benign Modbus traffic to reconstruct its input, so that traffic diverging from the benign distribution produces elevated reconstruction error and can be flagged as anomalous. Because the model is trained on benign traffic alone, it does not require labelled attacks for training; the labels from the orchestrator (Section 6) are used for evaluation, not for fitting the model.

The CNN-BiLSTM-AE architecture sits at the intersection of two requirements for Modbus anomaly detection on edge hardware: local transactional patterns (function-code sequences over a few timesteps, captured by the convolutional front-end) and session-level context (function-code transitions over longer horizons, captured by the bidirectional LSTM). Dense, LSTM-only, and Transformer-only alternatives each miss one of the two requirements or exceed the Jetson 8 GB parameter budget shared with the Ollama LLM process.

The model processes sequences of 10 timesteps, each with 8 features. The 10-timestep window covers 5 s at the standard 500 ms SCADA poll interval (ten Modbus transactions per window). Feature set:

- **Protocol features (4):** Function code, register count, payload-length deviation (against the Modbus specification per function code), request–response latency. These cover the protocol-visible quantities an anomalous Modbus interaction must disturb (scans, forced-output attacks, DoS, malformed payloads).
- **Temporal features (4):** Request rate, function-code read fraction (rolling 50-FC window per IP, ≈25 s at the same poll interval), function-code transition entropy (Shannon entropy $H\left(X\right)=-\sum_{i}p_{i}\log_{2}p_{i}$ over consecutive transition pairs), and time of day. These capture behaviour invisible at the single-flow level.

**Figure 3:**
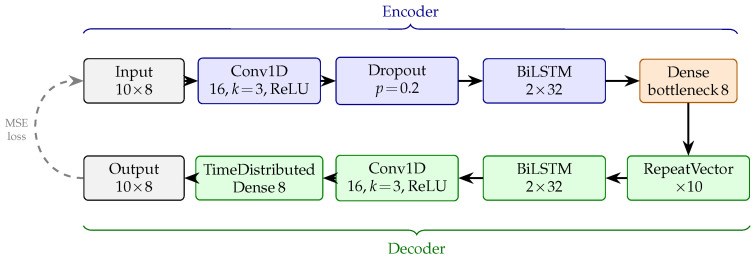
CNN-BiLSTM autoencoder used as the Modbus detector. The encoder (blue) compresses a 10 × 8 Modbus sequence (10 timesteps, 8 features) to an 8-unit dense bottleneck (orange) through one Conv1D layer, dropout, and a bidirectional LSTM. The decoder (green) mirrors the encoder via RepeatVector and a TimeDistributed dense output. Training minimises the per-sequence mean-squared reconstruction error against the input. The input and output blocks and the reconstruction-loss path are drawn in gray.

Architecture hyperparameters: Conv1D 16 filters, kernel 3, ReLU; dropout p=0.2; BiLSTM 32 units per direction; dense bottleneck 8 units, compressing the 10×8=80 input dimensions by an order of magnitude. Total parameter count is approximately 50,000, which fits the Jetson 8 GB shared budget alongside the concurrent Ollama LLM process. Figure 3 summarises the encoder–bottleneck–decoder layer stack. The promoted production model evaluated in Section 6.2 uses the eight input features listed above.

Training procedure

Training uses benign Modbus sequences only; the model learns to reconstruct normal patterns and produces elevated reconstruction error (mean-squared error, MSE) on out-of-distribution inputs. The autoencoder is trained on the benign partition of the labelled dataset (Section 6.2) with a held-out 10% validation split (4000 sequences) used for early stopping and threshold calibration; the attack partition is held out from training entirely. The encoder–decoder stack of Figure 3 is compiled with the Adam optimiser at learning rate $10^{-3}$ and the MSE loss, fitted in mini-batches of 32 sequences for up to 50 epochs. An early-stopping callback monitors the validation loss with patience 5 and restores the best-loss weights, which terminates training inside the 50-epoch budget on every run we have logged. The deployed thresholds ($\tau_{98}$, $\tau_{99.5}$) are calibrated post-hoc against the 98th and 99.5th percentiles of reconstruction errors on this held-out validation partition, targeting benign-side false-positive rates of ≈2% and ≈0.5% respectively. Confidence scores arriving at the SIEM index are produced by a min–max projection of the raw reconstruction error against two percentile anchors of the same held-out validation distribution: the median as the lower anchor and the 99.5th percentile as the upper anchor, with the result clipped to [0, 1]. A calibrated score of 0.5 therefore corresponds to the $\tau_{98}$ alerting threshold and a score of 1.0 saturates at the $\tau_{99.5}$ tail. The two anchor percentiles are stored alongside the model artefact so that downstream consumers read the calibration coefficients from the artefact rather than recomputing them per request. The promoted production model is a fine-tuned variant of this architecture (Adam at $10^{-4}$, 20 epochs, same early-stopping rule); the fine-tuning step dropped the register-address feature, which did not improve reconstruction quality, leaving the eight input features described above.

Training cost and deployment footprint

The ∼50,000-parameter network corresponds to a serialised model artefact under 1 MB on disk in single-precision float. Training is performed on a development workstation with a single NVIDIA RTX-class GPU (NVIDIA Corporation, Santa Clara, CA, USA) and TensorFlow 2.16; the 50-epoch budget over the 36,000-sequence benign training partition completes in the order of minutes on this hardware, with peak training-time memory bounded below 2 GB, and the early-stopping callback typically terminates inside the budget. The fine-tuning pass is a smaller second stage of 20 epochs against the same partition. At deployment the artefact is loaded onto a Jetson Orin Nano (8 GB shared SoC, ARM Cortex-A78AE); per-window inference tracks the steady-state SCADA poll rate (1–10 transactions per second per device pair) within the poll-cycle budget, so the detector is bounded by the Modbus poll cycle rather than by its own compute. Training and inference are not run concurrently on the Jetson because the LLM-assisted SIEM consumer co-resides on the same SoC and the 8 GB budget is shared; on-device retraining is operator-triggered and runs only when inference is paused. Production-class deployment in a real ICS environment would either follow the same train-off-device/deploy-on-device split used here or replace the Jetson with an x86 industrial gateway with more headroom, depending on the operator’s edge-compute envelope.

### 5.3. PROFINET Anomaly Detector

The PROFINET detector is an ExtraTrees classifier trained on 111 IAT-derived features extracted from fixed-length windows of PROFINET Real-Time (PN-RT) frames. ExtraTrees was selected over alternatives (Random Forest, XGBoost, a dense neural classifier) on three properties of the feature space: tree ensembles handle IAT features without requiring normalisation or distributional assumptions, which matters because cycle-time distributions differ across sites; the random-split criterion provides additional regularisation in the high-dimensional IAT feature space, reducing variance on small per-site training sets; and tree models are interpretable via feature importance, which enables per-site threshold calibration. The feature extractor operates in two stages: per-frame quantities (timing, cycle-counter behaviour, frame length, and status) are computed first, then aggregated to window-level features using five summary statistics (mean, standard deviation, 95th and 99th percentiles, and maximum). IAT statistics are the dominant predictive channel; non-IAT fields contribute marginally. The classifier detects any anomaly that perturbs cyclic timing; the same IAT deviation generalises across topology changes, device failures, and timing-based denial-of-service. We integrate the PROFINET detector into the protocol-aware pipeline through the router described below.

Methodology summary

The PROFINET detector trains on benign-only PN-RT capture (Table 1 caption); each capture covers a contiguous period of normal operation, and the 111-feature window vector is built from the per-frame quantities listed above, aggregated by the five window-level statistics. Per-site threshold calibration sets the operating point at ≈5% benign-side false-positive rate against the held-out portion of the same-site benign capture (Table 1, Threshold column). On a multi-site ground-truth corpus of 158 industrial PROFINET captures spanning 53 production lines and 53 confirmed ring-open anomalies, the ExtraTrees configuration used here achieves an area under the receiver operating characteristic curve of 0.967 (95% CI [0.94,0.99]), 88.7% true-positive rate at the 5% false-positive operating point, 92.2% precision, and $F_{1}=0.904$ under leave-one-line-out cross-validation [19]. We report the router-and-dispatch integration that places the PROFINET detector alongside the Modbus detector behind a single labelled-evaluation interface; the per-technique evaluation in Section 6 therefore exercises the Modbus path on the labelled Modbus partition only.

### 5.4. Protocol-Router Dispatch

The router invokes at most one detector per flow. A Modbus flow is scored only by the Modbus CNN-BiLSTM-AE; a PROFINET flow only by the PROFINET detector; flows classified as other (IT traffic, HTTP, MQTT, reconnaissance activity) bypass the ML stage and are routed to the SIEM rule layer. No cross-detector score fusion takes place at this stage; per-flow scored events are consumed by the downstream SIEM-based detection framework.

Table 1 summarises the detection model specifications.

The PROFINET detector is integrated into the testbed pipeline through the protocol router. The PROFINET ground-truth signal is cyclic-timing failure rather than MITRE-technique-labelled intrusion, so the per-technique evaluation in Section 6 uses the Modbus CNN-BiLSTM-AE on the labelled Modbus partition.

Adding a new protocol to the router

Extending the router to a third protocol (DNP3, OPC-UA, EtherCAT, IEC 61850, MQTT) is two changes against the same interface. First, one additional classification rule is added to the priority-ordered rule set above. The rule is a port-and-pattern match for the new protocol, inserted below the recon-token rule and above the default other branch, with the same top-down precedence semantics as the Modbus and PROFINET rules; a low-packet-count gate analogous to the one used for PROFINET applies wherever the protocol requires a minimum sample count to evidence its signature. Second, a per-protocol detector plug-in is registered against that classification. Any model whose input shape matches the plug-in’s training-time feature vector is admissible at this interface, independent of the rest of the pipeline. The labelling primitive (Section 4) is unchanged across this extension: it operates at injection time inside the orchestrator and is therefore protocol-agnostic. The Modbus and PROFINET plug-ins evaluated here are two instances of the same interface; the evidence offered for the dispatch logic itself is the routing of labelled flows to the correct plug-in across both protocols. A third protocol enters through the same two-change procedure above, against the same interface contract.

## 6. Evaluation

The evaluation produces two complementary artifacts: a per-technique detection result on the labelled Modbus dataset (Section 6.2), and a chain-level multi-stage validation on four CALDERA profiles (Section 6.3). The first is a controlled measurement against labelled ground truth; the second measures the pipeline output under realistic adversary execution, including adversary-side failure.

### 6.1. Experimental Setup

The detection pipeline runs on a Jetson Orin Nano (8 GB shared RAM, ARM Cortex-A78AE); SIEM rules run on a dedicated Elasticsearch deployment. The rule set, whose design and per-rule ATT&CK mapping are detailed in the companion framework paper [20], comprises 27 enabled Elastic Kusto Query Language (KQL) rules mapped to MITRE ATT&CK Enterprise and ICS, partitioned into IT-tagged rules (credential access, lateral movement, enterprise enumeration) and OT-tagged rules (Modbus DoS, safety-system tampering, forced-output manipulation, covert-channel signalling), with a small disabled-by-default partition of specification-ambiguous patterns. The 27 enabled rules emit the 30 alert events reported in Section 6.3; rule count and alert count refer to distinct quantities.

### 6.2. Labelled Modbus Dataset and Per-Technique Detection

The labelled dataset contains 40,000 benign Modbus sequences (collected during normal testbed operation across the two SCADA endpoints) and 9997 attack sequences distributed across four ATT&CK for ICS techniques. The autoencoder is trained on the benign partition with a held-out 10% validation split (4000 sequences) used for early stopping and threshold calibration (Section 5, Training procedure); the 9997 attack sequences are held out from training entirely and consumed only at evaluation time. The benign sequences were captured in orchestrator-controlled time windows with no overlapping attack execution, and the attack sequences in subsequent separate windows with the orchestrator emitting the labelled attack sequences, so benign and attack samples occupy disjoint capture windows and share no overlapping packet trace. The Modbus detector evaluated here is the production deployment of the protocol-aware pipeline described in Section 5; thresholds and reported numbers correspond to the live promoted model.

**Definition** **1.** *Two thresholds are reported on the same model. The 98th-percentile threshold ($\tau_{98}=1.263$) and the 99.5th-percentile threshold ($\tau_{99.5}=1.345$) are calibrated at the corresponding percentiles of benign reconstruction errors on the autoencoder’s held-out validation partition and are reused unchanged at evaluation. TPR in Table 2 is the per-technique fraction of attack samples whose reconstruction error exceeds the threshold. FPR is the fraction of held-out benign validation sequences above the threshold; Precision and $F_{1}$ in Table 3 are computed on the combined held-out partition (4000 benign validation sequences and the 9988 windowed attack sequences of Table 2), with recall equal to the TPR*.

On the labelled corpus, the Modbus autoencoder reaches 100% TPR at $\tau_{98}$ on all four techniques and a 99.7% overall TPR at $\tau_{99.5}$. Per-technique TPR at $\tau_{99.5}$ stays high: T0802 Collection and T0846 Remote System Discovery hold at 1.000, T0831 Manipulation of Control at 0.995, and T0836 Modify Parameter at 0.961. Table 3 reports the operational metrics at both thresholds: precision stays above 0.99 and the benign-side false-positive rate is fixed by construction at 2.0% ($\tau_{98}$) and 0.5% ($\tau_{99.5}$). This is the per-technique discrimination that coarse-labelled public datasets cannot support: the dataset allows a detection author to state which techniques their method handles at which false-positive cost.

**Figure 4:**
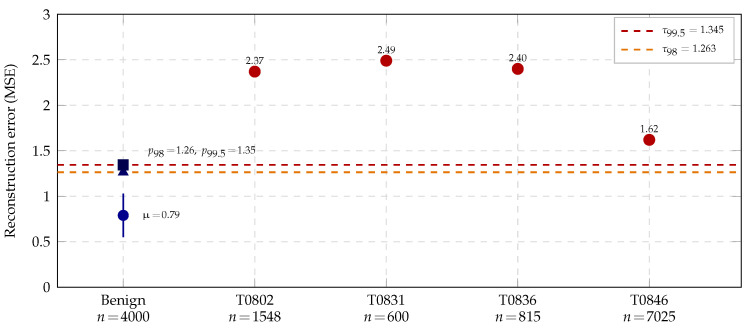
Per-class reconstruction-error summary on the labelled evaluation corpus. The benign column shows the empirical mean (filled circle) and μ±σ vertical span of the validation distribution; its 98th and 99.5th percentiles coincide with the calibrated thresholds (triangle/square markers on the dashed lines). The four attack columns show per-technique mean errors. Dashed horizontal lines mark the calibrated thresholds $\tau_{98}=1.263$ and $\tau_{99.5}=1.345$. All four attack-class means sit above $\tau_{99.5}$, consistent with the per-technique TPR at or above 0.961 in Table 2.

Benign and attack reconstruction errors separate cleanly on the labelled partition. Benign validation sequences concentrate at mean 0.79 (σ 0.24); the 98th and 99.5th percentiles of the validation reconstruction-error distribution are the calibrated thresholds $\tau_{98}=1.263$ and $\tau_{99.5}=1.345$. Mean attack errors are 2.37 (T0802), 2.49 (T0831), 2.40 (T0836), and 1.62 (T0846); all four per-technique means clear $\tau_{99.5}$, which is why per-technique TPR at $\tau_{99.5}$ stays at or above 0.961 in Table 2. Figure 4 plots these per-class summaries against the two thresholds.

**Scope of the headline number.** The campaign orchestrator both emits the attack sequences and writes their per-sequence technique labels, and the autoencoder is evaluated on the labelled partition produced by that orchestrator. Three factors bound the resulting risk: (i) the benign-vs-attack reconstruction-error margin reported above (benign validation mean 0.79 vs. per-technique attack means 1.62–2.49, with all four attack means clearing $\tau_{99.5}$); (ii) the benign-side false-positive rates fixed by construction at the 98th and 99.5th percentiles of the benign validation distribution (2.0% and 0.5%, Table 3); (iii) the attacker-profile assumption acknowledged in Section 7.2. Cross-validation against attack traffic from a tool outside the labelling stack is the strongest remaining control, listed as the immediate next research step in Section 7.2.

**Statistical surface.** Per-technique TPR at the two thresholds is reported in Table 2; the operational metrics (TPR, FPR, precision, $F_{1}$) and the binary confusion counts (TP, FN, FP, TN) at both thresholds are reported in Table 3, computed on the combined held-out partition. Because each rate is a proportion measured on a finite held-out sample, we report a 95% Wilson score confidence interval for each: TPR is 1.000 [0.9996, 1.0000] at $\tau_{98}$ and 0.997 [0.9957, 0.9979] at $\tau_{99.5}$; the benign-side FPR is 2.0% [1.6%, 2.5%] and 0.5% [0.32%, 0.77%]; and precision is 0.992 [0.990, 0.994] and 0.998 [0.997, 0.999]. The intervals are narrow because the held-out partition is large (9988 attack and 4000 benign sequences), so the reported operating points are statistically tight. Recall equals the TPR column; precision stays above 0.99 at both thresholds, and $\tau_{99.5}$ trades 0.003 of recall for a four-fold reduction in benign-side false-positive rate. The benign-vs-attack margin in Figure 4 shows that this separation is a property of the reconstruction-error distributions and not of the threshold placement: all four attack-class means clear $\tau_{99.5}$.

**Threshold sweep and baseline comparison.** Table 3 reports the detector at its two deployed operating points, $\tau_{98}$ and $\tau_{99.5}$, which are the two decision-relevant rows of a receiver-operating-characteristic curve for a fixed-threshold deployment. Across these two points, the headline TPR remains saturating (≥0.997) while the benign-side FPR varies four-fold (2.0% to 0.5%); a tighter sweep that resolves additional percentiles (for example $\tau_{95}$, $\tau_{99}$, $\tau_{99.9}$) would trace the operating curve between the two reported points but cannot raise the TPR above the saturated value visible at $\tau_{98}$. A head-to-head comparison against a generic-flow baseline detector (for example, an autoencoder over NetFlow five-tuple aggregates) trained on the same labelled corpus would quantify how much of the per-technique TPR is attributable to per-protocol features rather than to orchestrator-specific regularities. We do not run that baseline on the present corpus, so the discussion of feature-engineering policy in Section 7.3 is framed as a design rationale rather than as a measured comparison. Both extensions, a full threshold sweep on the deployed model and a generic-flow baseline on the same labelled corpus, are listed alongside the external-tool cross-validation as the immediate next evaluation steps in Section 8.

### 6.3. Multi-Stage Validation via CALDERA Chains

In addition to the per-technique dataset, we report results for the four chains specified by the campaign profiles of Section 4 (each profile is the YAML artefact; each chain is its end-to-end execution). These chains serve here as a multi-path probe of the pipeline, showing that the testbed produces labelled telemetry along each detection path (ML+SIEM, ML-dominant, ML-only, SIEM-only). For each chain, we report: *Modbus detections*, the number of Modbus-layer sequences that the CNN-BiLSTM-AE flagged as anomalous within the chain’s wall-clock window; *SIEM alerts*, the number of Elastic rule matches that fired in the same window; *per-chain tactic coverage*, the fraction of executed CALDERA-ability tactics with at least one matching detection event; and *CALDERA-ability success rate* (*Cald. succ.*), the fraction of abilities in a chain profile that executed to completion, read from the CALDERA operation records.

The four chains exercise complementary paths through the detection pipeline. Chain 1 produces both ML evidence (92 Modbus detections from the write payload) and SIEM evidence (12 IT-tagged alerts from the lateral-movement phase). Chain 2 is ML-dominated (50 detections, 4 alerts) because the persistence-heavy path spends more time on OT modification than on SIEM-flagged IT actions. Chain 3 is ML-only (92 detections, 0 alerts): T1003 (OS Credential Dumping) is outside the enabled IT-tagged rule set deployed against this testbed, and the Modbus autoencoder fires on the subsequent PLC pivot. Chain 4 is SIEM-only (0 detections, 14 alerts): the chain stays on the IT side and exercises the IT rule coverage (T1087.002, T1482, T1484.001, T1098) without reaching Modbus, which is the complementary design case. The per-chain tactic coverage denominator is the set of distinct MITRE tactics represented by the abilities attempted in the chain profile; a tactic counts as covered if at least one detection event (model or SIEM) matches an ability of that tactic during the chain’s execution window. The CALDERA-ability success rate is the fraction of abilities in a chain profile that executed to completion, read from the CALDERA operation records for each chain.

Interpreting the chain detection counts

The Modbus-detection counts in Table 4 are reported over each chain’s full wall-clock execution window and are used here as a multi-path probe rather than as a precision metric: they show which detection path each chain exercises (Chain 1 ML and SIEM, Chain 2 ML-dominant, Chain 3 ML-only, Chain 4 SIEM-only). The quantitative per-technique detection evidence, where each attack sequence carries its MITRE technique label and the detector is reported at its two calibrated operating points, is the labelled-corpus evaluation of Table 2 and Table 3. Chain 4’s 0 Modbus detections against 14 SIEM alerts is the complementary case: an enterprise-only path produces no Modbus traffic to score.

Failure modes of the unsuccessful abilities

The 14–20% of abilities marked as not-completed across the four chains fall into three causes, identified by inspecting the CALDERA operation records: (i) *environmental* failures, where an ability assumes a host artefact that is not present on the target (for example, a credential-spray ability that probes accounts not provisioned in the engineering-workstation user store, or a Group-Policy ability that targets an Organisational Unit (OU) that does not exist), accounting for the largest share; (ii) *timeout* failures, where Sandcat reports the ability as launched but the result-collection deadline expires before output is captured (most frequent on the Linux-PLC pivot in Chain 3 over the slower OT VLAN); and (iii) *platform-fit* failures, where an ability bundled in CALDERA’s enterprise plug-in is registered for a platform whose binary is unavailable on the testbed image (for example, a Windows-only privilege-escalation step attempted by Chain 4 on a Linux fall-back agent). None of the failures are MITRE-technique-specific; they are environment-dependent and resolved either by adjusting the campaign profile to the testbed’s host inventory or by extending the result-collection window. Reporting the rate alongside the detection counts (Table 4) keeps the denominator transparent: detection counts are over the abilities that executed to completion, not over the profile size.

Per-tactic coverage detail

The aggregate per-chain tactic coverage in Table 4 (0.714–0.786) decomposes by tactic class as follows. ATT&CK Enterprise tactics on the IT side (Initial Access, Credential Access, Discovery, Lateral Movement, Persistence) are covered whenever the chain reaches them, because the enabled IT-tagged rules of the SIEM rule set carry detection rules for the corresponding ability families (Chain 1 produces 12 alerts under Initial Access and Lateral Movement; Chain 2 produces 4 under Credential Access and Persistence; Chain 4 produces 14 under Discovery and Persistence). ATT&CK for ICS tactics on the OT side (Collection, Inhibit Response Function, Impair Process Control, Impact) are covered whenever the Modbus path is exercised, because the autoencoder fires on the labelled Modbus interactions corresponding to those abilities (Chains 1–3 produce 234 Modbus detections in aggregate, distributed across T0888/T0861/T0836/T0806). The uncovered tactics within each chain concentrate in two categories: (i) Execution and Lateral Movement abilities whose payload binary is unavailable on the testbed image (the platform-fit failures above), and (ii) one or two Persistence abilities whose corresponding SIEM rule is in the disabled-by-default partition of specification-ambiguous patterns documented in the companion framework paper [20]. Neither category is methodology-correlated; both close with engineering changes to the testbed image and the rule-enablement profile rather than with changes to the labelling primitive or the router.

## 7. Discussion

This section reviews the evaluation results in the frame introduced in Section 1: one primary contribution (the labelling methodology) and two supporting contributions (the four CALDERA chains and the protocol-aware detection pipeline) demonstrated on one use case. Section 7.1 reports the headline measurements produced by the use case described in Section 3, Section 4 and Section 5: per-technique Modbus detection rates and multi-stage chain telemetry. Section 7.2 records the properties of the use case that bound the inferences supported by those measurements; cross-use-case evaluation along the same axes (other testbeds, other protocols, other detector plug-ins) is named as the next use case in Section 8. Section 7.3 draws design observations that follow from the methodology’s load-bearing property and the router’s load-bearing architectural choice.

### 7.1. Key Findings

The per-technique evaluation on the MITRE-mapped Modbus attack dataset is the lead finding: the live Modbus autoencoder reaches 100% TPR at $\tau_{98}$ across all four labelled techniques (T0802, T0831, T0836, T0846) and a 99.7% overall TPR at the tighter $\tau_{99.5}$ threshold. Per-technique TPR at $\tau_{99.5}$ stays between 0.961 (T0836 Modify Parameter) and 1.000 (T0802 Automated Collection and T0846 Remote System Discovery); all four per-technique attack-class means clear $\tau_{99.5}$, so the headline numbers reflect a clean benign-attack separation on this corpus. The fact that these numbers can be reported *per technique*, rather than only as an aggregate, is the value that in-orchestrator labelling adds over coarse-labelled public datasets. A Modbus IDS evaluated on a corpus built with this labelling methodology can state exactly which techniques it handles and at what false-positive cost, rather than reporting only an aggregate detection rate.

The multi-stage chain evaluation provides complementary evidence that the testbed also supports end-to-end kill-chain generation, not just isolated-technique injection. The four evaluated chains yield 234 Modbus-layer detections and 30 SIEM alerts with per-chain tactic coverage between 0.714 and 0.786 and CALDERA-ability success rates between 0.800 and 0.857, and they exercise four complementary detection paths (ML + SIEM, ML-dominant, ML-only, SIEM-only) whose contrasting outputs make each layer’s coverage directly inspectable. The main aim of the chain evaluation is to show that the testbed can produce diverse IT-to-OT attack progressions whose per-sequence technique labels feed the labelled dataset.

The protocol-router dispatch (Section 5) prevents inapplicable models from scoring a flow that does not match their training distribution, which is the mechanism we credit for keeping per-technique false positives low on the Modbus-labelled partition.

### 7.2. Limitations

The bullets below name the properties of the use case that bound the inferences supported by the headline measurements; in the methodology-vs-use-case frame, each bullet identifies an axis along which a future use case of the methodology would extend the evidence.

- **Use-case hardware fidelity, scale, and site count.** The use case uses Raspberry Pi PLCs, fewer than 20 devices, and a single deployment site; Section 7.4 separates what this exercises directly, what it approximates, and what it does not test. A use case built on production PLCs at a partner site is the path that extends the hardware-fidelity evidence.
- **Use-case attacker profile.** The per-technique TPRs assume the CALDERA emission profile of the use case (distinct function-code patterns, register-addressing signatures, timing footprints); adversarially crafted traffic mimicking benign Modbus patterns is not captured. A use case whose label primitive is hosted by a second emulator (Atomic Red Team, a hand-written Modbus injector) is the path that quantifies the share of the headline TPR attributable to orchestrator-specific regularities versus per-protocol semantics.
- **Use-case training distribution.** Benign training data for the Modbus detector comes from one testbed; the labelling primitive itself is hardware-independent at the orchestrator interface. A use case whose benign capture is sourced from an independent OT deployment is the path that quantifies cross-site benign-distribution drift.
- **Headline saturation on the use case.** A 100% TPR at $\tau_{98}$ on every evaluated technique, and a 99.7% overall TPR at $\tau_{99.5}$, characterises this use case: orchestrator-driven attack emission against commodity Raspberry-Pi Modbus servers, with the Modbus detector trained on the benign capture from the same testbed. These numbers are a per-use-case calibration result, not a universal TPR claim: a use case whose attacker profile or PLC substrate departs from this one is expected to report a different operating point on the same calibration protocol, and the threshold pair ($\tau_{98}$, $\tau_{99.5}$) is the handle an operator re-derives per deployment.

### 7.3. Operational Implications

Two design observations follow from the methodology’s load-bearing property and the router’s load-bearing architectural choice, and apply to other use cases. First, the router architecture (the detection pipeline’s load-bearing choice) admits two coherent feature-engineering policies: a per-protocol plug-in (the use case here, trained on Modbus function codes, register patterns, and inter-arrival-time statistics) and a generic-flow plug-in (a detector trained on NetFlow five-tuple aggregates over the same labelled corpus). The two policies share the labelled-evaluation interface produced by the labelling primitive, so the choice between them is a feature-engineering decision at the plug-in interface and a comparable use case of the same router; this paper instantiates the per-protocol policy because protocol-aware features expose Modbus-specific anomaly classes (function-code misuse, malformed register access, MEI-based reconnaissance) that are not visible at the IP/port flow level, but it does not claim that the per-protocol policy dominates the generic-flow policy as a measurement on this paper’s data: each plug-in is a separate use case and its TPR/FPR profile is a per-use-case calibration question. Second, the edge deployment evaluated here (a Jetson Orin Nano running a Modbus CNN-BiLSTM-AE of ∼50,000 parameters) fits inside the host SoC’s resource budget for this use case; use cases built on other shared-memory edge devices of comparable capacity inherit the same router architecture and labelling interface and re-calibrate the per-use-case latency budget against their host.

### 7.4. Constraints of Real-Industrial Deployment

Industrial deployments impose constraints that this testbed approximates rather than reproduces. We separate them by what the current evaluation does test, what it approximates, and what it cannot test on the present hardware.

**Tested directly.** Per-protocol Modbus and PROFINET feature extraction (function codes, register-address structure, inter-arrival-time distributions) on a physical OT network with real switches and the multi-layer monitoring stack described in Section 3.

**Approximated.** Deterministic OT timing and industrial jitter: the testbed uses Raspberry Pi devices in place of production PLCs, so request–response latency is in the order of milliseconds rather than the sub-millisecond range typical of industrial controllers, and timing-based detection features may behave differently on production hardware (Section 7.2, PLC fidelity bullet). Fieldbus synchronisation is exercised at protocol level (PROFINET cycle structure, Modbus poll cycles) but without the hard real-time guarantees of an industrial process loop. Network scale is approximated at fewer than 20 devices across two VLANs; multi-zone industrial environments would exhibit additional traffic patterns the present evaluation does not capture.

**Not tested.** PLC firmware behaviour, vendor-proprietary protocol extensions, hardware interrupts, and embedded safety-system responses are tied to specific vendor stacks not present in the testbed. The labelling methodology and the protocol router are protocol-agnostic at their interface, so extending evaluation to a deployment that includes these layers is an engineering integration, not a methodological re-design.

Cross-site evaluation against a deployment that includes production PLCs and vendor-specific firmware is the natural next step for closing each of the three groups above.

## 8. Conclusions

We described one primary contribution and two supporting contributions, demonstrated on one operational use case. The *primary* contribution is an in-orchestrator labelling methodology for per-technique-labelled ICS attack capture, with one load-bearing property: the campaign orchestrator owns the label primitive and writes each emitted attack sequence’s MITRE ATT&CK for ICS technique identifier into the capture artefact at injection time, eliminating ex-post alignment of operation logs against packet timestamps. The labelling interface is portable across emulators (CALDERA in this use case, with Atomic Red Team and MITRE Engenuity Center for Threat-Informed Defense as direct equivalents) and across fieldbus protocols (a Modbus PCAP-plus-JSONL pairing in this use case, with PROFINET, DNP3, OPC-UA, EtherCAT, and IEC 61850 captures as protocol equivalents that the labelling interface absorbs without change). The *first supporting* contribution is a protocol-aware detection pipeline whose load-bearing architectural choice is a priority-ordered protocol router that dispatches each labelled flow to a per-protocol detector plug-in (a Modbus CNN-BiLSTM autoencoder over per-protocol features in this use case, with a generic-flow autoencoder over NetFlow aggregates as a comparable plug-in policy on the same router). The *second supporting* contribution is the four reproducible CALDERA chains that exercise the labelling methodology end-to-end and the pipeline along complementary detection paths. Practitioners running a different emulator, a different fieldbus protocol, or a different detector can apply the methodology’s load-bearing property and the router’s architectural choice; the Modbus/Raspberry-Pi-PLC/CALDERA/CNN-BiLSTM combination reported here is the form of the evidence, not the scope of the contributions.

On the use case, the Modbus autoencoder reaches 100% TPR at $\tau_{98}$ across all four labelled techniques and a 99.7% overall TPR at $\tau_{99.5}$ (per-technique TPR between 0.961 and 1.000), with all four attack-class means clearing $\tau_{99.5}$. The four CALDERA chains produced 234 Modbus-layer detections and 30 SIEM alerts with per-chain tactic coverage between 0.714 and 0.786 and CALDERA-ability success rates between 0.800 and 0.857. These numbers calibrate the use case; what the methodology and pipeline together contribute is the per-use-case calibration protocol, not a universal TPR claim that propagates to other use cases.

The in-orchestrator labelling methodology produces per-technique-labelled corpora that support technique-resolved benchmarking of Modbus IDS methods; the corpus reported here is one instance, governed by the project consortium agreement recorded in the Data Availability Statement. A corpus produced by this methodology inherits the fidelity boundary of its substrate: the corpus here is generated against Raspberry Pi servers running open-source Modbus stacks and does not reproduce the sub-millisecond latency, cycle-jitter, or vendor-firmware quirks of production controllers. A method evaluated on such a corpus measures behaviour against orchestrator-emitted traffic on commodity Modbus servers, not against production OT traffic, and should be reported with that scope. Each future use case (different emulator, different protocol, different detector plug-in, different PLC substrate) extends the evidence base along the axis it varies.

Boundaries on the evidence base

Three boundaries shape what the headline numbers above say, and the reader should hold them in view alongside the numbers. First, the labelled corpus spans four ATT&CK for ICS techniques (T0802, T0831, T0836, T0846) rather than the full taxonomy; the four were selected as a cross-section of collection, manipulation, and discovery techniques sized to instantiate the labelling methodology end-to-end on a coherent attack family. Second, the per-technique detection numbers are reported on the Modbus plug-in instance of the router; PROFINET coverage is established through the methodology summary of Section 5 and the multi-site leave-one-line-out evaluation in the companion paper [19], and additional fieldbus protocols enter through the plug-in contract documented in the same section. Third, the labels are written by CALDERA at injection time, which conditions the per-technique TPR on a single orchestrator’s emission profile and on the regularities of one labelling stack; adversarially crafted traffic mimicking benign Modbus polling and attack traffic generated outside the labelling stack are not represented in the corpus. Each boundary names a research-program axis along which a future use case extends the evidence: a broader technique selection on the first axis, a second fieldbus plug-in or a generic-flow plug-in on the second, and an external-tool attack source on the third. The methodology, the router architecture, and the inline calibration protocol are invariant across all three: the labelling primitive operates at the orchestrator interface regardless of which emulator or fieldbus a use case selects, and the router’s plug-in interface admits any detector whose input shape matches its training-time feature vector. The future work directions below extend the evidence along these axes in the same order.

Future work will focus on three directions. First, cross-validation against attack traffic generated by a tool outside the labelling stack (hand-written Modbus injectors, alternative emulation frameworks), to quantify how much of the current margin is attributable to per-protocol semantics versus orchestrator-specific regularities. Second, deployment in industrial partner environments to validate detection performance on production OT networks with real PLCs and physical processes; this is also the path that closes the PLC-fidelity boundary inherited by the dataset. Third, extending the testbed’s protocol coverage to OPC-UA, EtherCAT, DNP3, and IEC 61850, following the precedent set for Modbus and PROFINET. A separate use case using a generic-flow plug-in on the same router architecture is the natural companion experiment to the per-protocol use case reported here. A fuller statistical characterisation of the Modbus detector, with repeated-run confidence intervals, full ROC and per-technique distributional analysis, and robustness under injected traffic noise, is a further evaluation direction. Federated learning for distributed anomaly detection across multiple deployment sites remains an open direction.

## Figures and Tables

**Table 1:** Detection model specifications. The Modbus autoencoder is trained on benign traffic only and flags anomalies via reconstruction error (mean-squared error above the calibrated threshold), with operating thresholds set at the 98th and 99.5th percentiles of reconstruction errors on a held-out benign validation partition. The PROFINET ExtraTrees classifier is trained on benign-only PN-RT capture and flags anomalies by classification on 111 inter-arrival-time-derived window features, with the operating point calibrated to ≈5% benign-side false-positive rate against held-out same-site benign capture. Parameter counts are verified against the model definitions in the project repository.

Model	Architecture	Features	Trainable Params	Threshold
Modbus AE	CNN-BiLSTM-AE	8	∼50,000	98th/99.5th pctl
PROFINET	ExtraTrees (IAT features)	111	domain-tuned	Domain-calibrated (5% FPR)

**Table 2:** Per-technique detection performance of the Modbus CNN-BiLSTM-AE on the MITRE-mapped Modbus attack dataset produced by the testbed (40,000 benign + 9997 attack Modbus sequences). Thresholds are calibrated at the 98th and 99.5th percentiles of benign reconstruction errors on the autoencoder’s held-out validation partition ($\tau_{98}=1.263$, $\tau_{99.5}=1.345$). The per-technique rows sum to 9988 attack sequences after the 10-timestep sliding window; 9 sequences are absorbed by the window edge. *Mean error* is the per-technique attack-class mean reconstruction error. The bold row reports the overall (aggregate) figures across all four techniques.

Technique	MITRE ID	*n*	TPR @$\tau_{98}$	TPR @$\tau_{99.5}$	Mean Error
Automated Collection	T0802	1548	1.000	1.000	2.37
Manipulation of Control	T0831	600	1.000	0.995	2.49
Modify Parameter	T0836	815	1.000	0.961	2.40
Remote System Discovery	T0846	7025	1.000	1.000	1.62
**Overall**	n/a	**9988**	**1.000**	**0.997**	n/a

**Table 3:** Operational metrics and binary confusion counts of the Modbus CNN-BiLSTM-AE at the two thresholds, on the combined held-out partition (4000 benign validation sequences and the 9988 windowed attack sequences of Table 2). True positives (TP) and false negatives (FN) partition the 9988 windowed attack sequences; false positives (FP) and true negatives (TN) partition the 4000 benign validation sequences. The benign-side false-positive rate (FPR) is fixed by construction at the chosen percentile of the benign validation reconstruction-error distribution. Precision =TP/(TP+FP) and $F_{1}=2\;\mathrm{TP}/\left(2\;\mathrm{TP}+\mathrm{FP}+\mathrm{FN}\right)$. A 95% Wilson score confidence interval for each rate metric is reported in the text.

Metric	$\tau_{98}=1.263$	$\tau_{99.5}=1.345$
True positives (TP)	9988	9958
False negatives (FN)	0	30
False positives (FP)	80	20
True negatives (TN)	3920	3980
True-positive rate (TPR)	1.000	0.997
False-positive rate (FPR)	2.0%	0.5%
Precision	0.992	0.998
$F_{1}$	0.996	0.997

**Table 4:** Detection counts across four CALDERA kill chains, from the campaign evaluation log of the 26 March 2026 run. The *Cald. succ.* column is reported for denominator transparency. The bold row reports the aggregate across all four chains.

Chain	Abilities	Cald. Succ.	Modbus Det.	SIEM Alerts	Coverage
1 (phish + lateral + Modbus write)	11	0.818	92	12	0.714
2 (password spray + persistence)	11	0.818	50	4	0.786
3 (Linux cred theft, PLC target)	5	0.800	92	0	0.786
4 (domain escalation via GPO)	14	0.857	0	14	0.786
**Aggregate (4 chains)**	**41**	**0.829**	**234**	**30**	**0.768**

## Data Availability

Restrictions apply to the availability of these data. The data presented in this study were obtained within the BMWE-funded KISTE project (grant number KK5189606RG4) and are subject to a non-disclosure agreement between the project partners. They are not publicly available.
